# Notes and News

**Published:** 1903-03-14

**Authors:** 


### Notes and News.

### HOSPITAL MEETINGS AND FESTIVALS.

Royal Eye Hospital.—Annual General Meeting, Wednesday, March 18, at 4 p.m.

University College Hospital.—Annual General Meeting, Thursday, March 19, at 4 p.m.

Royal National Hospital for Consumption.—Annual General Meeting, Thursday, March 19, at 4.30 p.m., at the office, 30 Craven Street.

Royal Hospital for Diseases of the Chest.—Annual General Meeting, Thursday, March 19, at 3 p.m.

The Marquis of Bute has promised £1,000 to the Cardiff Infirmary, that it may be equipped with electric light.

The Lord Howard de Walden will preside at a festival dinner in aid of the funds of the Metropolitan Hospital, to be held at the Whitehall Rooms on Wednesday, May 20th, next.

The March number of the "World's Work" contains an illustrated description of the new consumption hospital at Beelitz, near Berlin. This hospital, which is also a convalescent home, is the largest in the world for similar cases.

A new out-patient department has been opened in connection with the Hospital for Children in Sydney, New South Wales. It is situated apart from the hospital, and has its own operating theatre, besides the usual offices and waiting-hall for two hundred. It is close to railway and tramcars.

Though the small-pox epidemic in Manchester shows some abatement the surrounding districts continue to report fresh cases. It has been proposed that a joint hospital should be opened, possibly in the vicinity of Wigan. There are a certain number of cases distributed throughout the North and West, in Northumberland, Staffordshire, Yorkshire, Cheshire, Derbyshire, Warwickshire, Herefordshire, Shropshire, and Wales.

There are still large numbers of plague cases in Bombay city and district, and a good deal elsewhere. The most recent bad outbreak is reported from Poona. Only a few cases are now in South Africa, and the same is the case in Hong Kong and Mauritius.

The County Council propose to erect another lunatic asylum, and in their scheme they have the concurrence of the Lunacy Commissioners. There is a need of further accommodation, and the recent occurrence at Colney Hatch has evidently shown the advisability of abolishing the temporary wooden buildings which contained the overflow from permanently-built asylums.

Railway accidents are so serious in America that it is not surprising that a suggestion has been made that ether and chloroform should be part of each train's equipment. The practical difficulty of administering either, however, has so far not been taken into consideration by those who have proposed it. A more practical suggestion is that every railway passenger should carry morphine with him.

No doubt general practitioners regard the recommendation of the London County Council in respect to their attendance at post-mortem examinations at inquests as the thin end of the wedge to abolish their services altogether. They propose having at call skilled pathologists ready to attend inquests when requested, but it is recommended that the regulations should provide for the attendance of the practitioners who have attended on persons on whom inquests are held whenever the coroner is satisfied that their evidence will be material.

A new lunatic asylum has been opened at Talgarth for the counties of Brecon and Radnor. The patients who properly belong to these districts will shortly be removed into it from the Monmouthshire Asylum. The accommodation is for 350 persons. The grounds occupy 27 acres, upon seven of which the buildings are erected. These are divided into six blocks, with further houses and buildings for administrative purposes. The cost has been about £430 per bed, but provisions for an inexpensive addition to the accommodation will ultimately bring this figure lower.

